# Minimizing bleeding risks during gastric neuroendocrine tumor endoscopic submucosal dissection by pre-emptive EUS-guided epinephrine injection

**DOI:** 10.1016/j.vgie.2025.04.010

**Published:** 2025-05-02

**Authors:** Radhika Chavan, Vishal Seth, Zaheer Nabi, Dadasaheb Maindad, Harshwardhan Dongre, Sanjay Rajput, Akhil Nagpal

**Affiliations:** 1 Department of Gastroenterology and Advanced Endoscopy, Bharati Vidyapeeth (deemed university), Pune, Maharashtra, India; 2 Department of Advanced Endoscopy, AIG, Hyderabad, Telangana, India; 3 Department of Gastroenterology and Advanced Endoscopy, Ansh Clinic, Ahmedabad, Gujrat, India

## Abstract

**Background and Aims:**

Gastric neuroendocrine tumors (NETs), although rare, are highly vascular subepithelial lesions that can pose significant bleeding risks during endoscopic submucosal dissection (ESD). Traditionally, bleeding is managed intraoperatively with mechanical or thermal hemostasis, but pre-emptive strategies remain underexplored. Here, we report the use of EUS-guided pre-emptive epinephrine injection to minimize the bleeding risk during gastric NET ESD.

**Methods:**

To minimize the risk of bleeding during ESD of large gastric NETs, a pre-emptive EUS-guided epinephrine injection was administered at the base of the lesion near the feeder vessel.

**Results:**

A 38-year-old female was diagnosed with a large gastric subepithelial lesion during evaluation for upper gastrointestinal bleeding. EUS revealed a large hypoechoic tumor confined to the submucosa with a prominent feeding vessel. Given the predominantly submucosal location, ESD was planned. However, significant bleeding was anticipated due to the large feeder vessel. Therefore, a pre-emptive EUS-guided adrenaline injection (5 mL of 1:10,000 diluted epinephrine) was administered at the base of the lesion after confirming absence of blood return on fine-needle aspiration. Instantaneous pallor of the lesion was observed. ESD was subsequently completed successfully without any bleeding.

**Conclusions:**

This case highlights a novel, effective, and safe use of EUS-guided pre-emptive epinephrine injection to minimize bleeding during ESD of vascular gastric NETs. This approach could enhance procedural safety and warrants further prospective evaluation.

## Case Report

**Figure 1:**
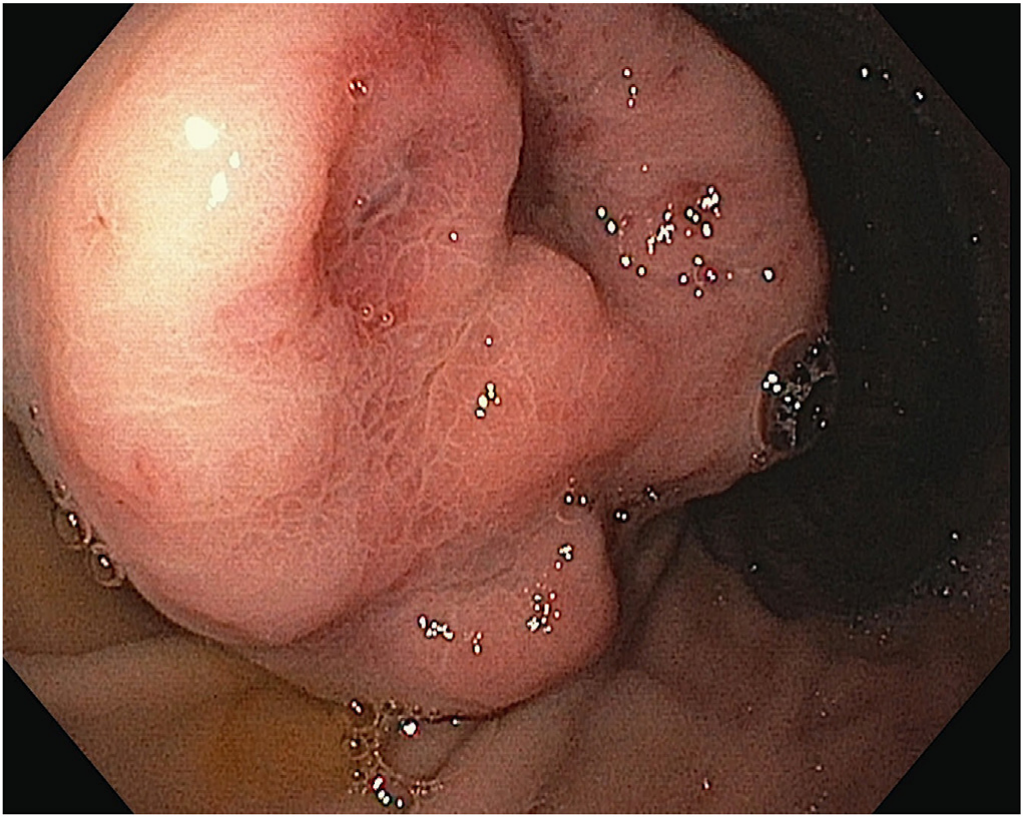
Gastroscopy showing a large subepithelial lesion with central superficial ulceration and a pigmented spot.

**Figure 2:**
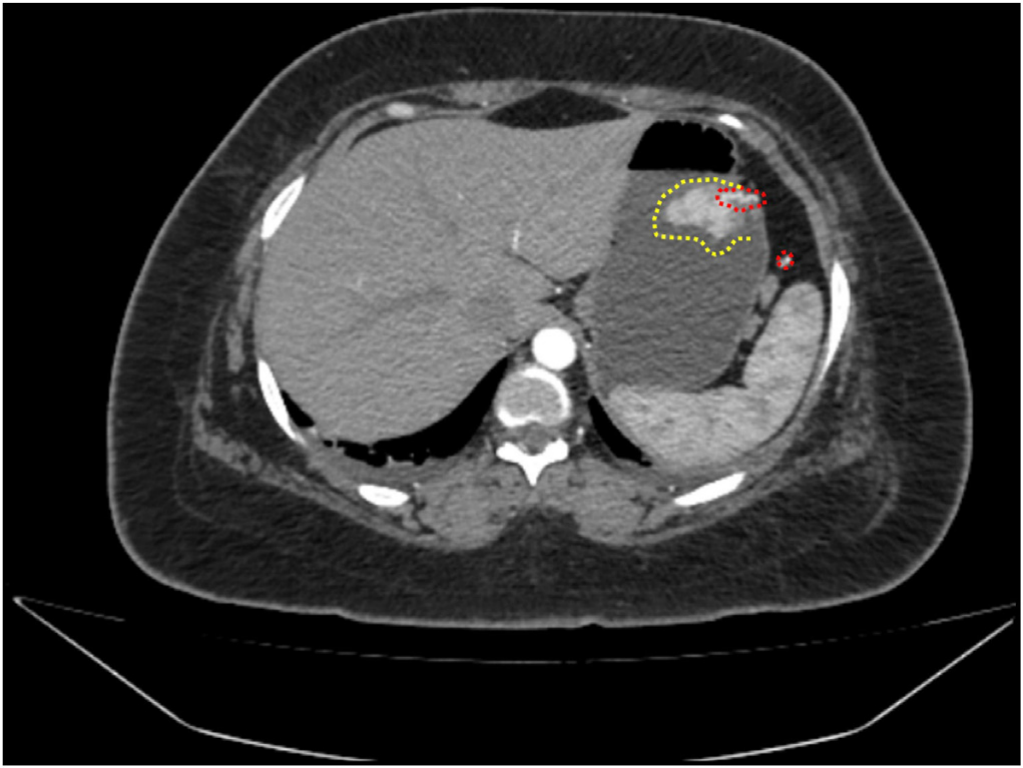
Contrast-enhanced computed tomography of the abdomen shows a large arterial enhancing lesion (*yellow dotted line*) in the body of the stomach along the greater curvature with feeder vessel arising from the short gastric branch (*red dotted line*).

**Figure 3:**
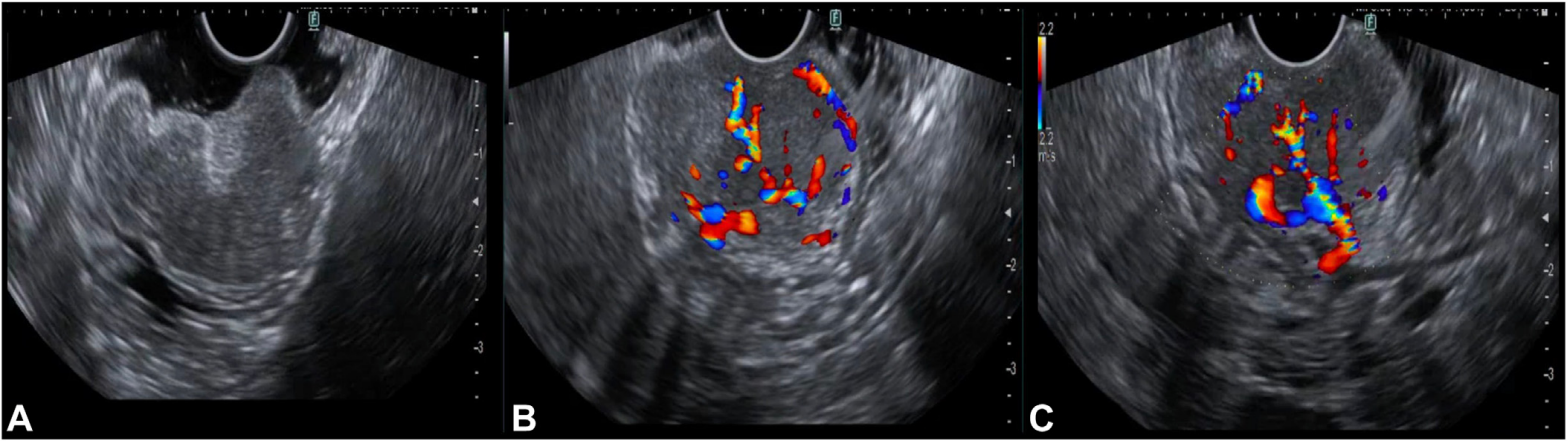
EUS view of the gastric subepithelial lesion. **A,** EUS revealed a large hypoechoic lesion with an irregular luminal surface seen within the submucosa, with focal infiltration into muscularis propria. **B,** The lesion shows diffuse vascular signal on the color Doppler. **C,** A large feeder vessel seen on EUS during color Doppler view.

A-38-year-old woman presented with hematemesis for 2 days. On examination, she appeared pale with mild tachycardia, but her blood pressure was within normal range. Her hemoglobin was 4.2 g/dL. She received 3 packed red blood cells. After stabilization, gastroscopy revealed a large subepithelial lesion (3-4 cm) along the greater curvature of the stomach, with a central ulceration and pigmented spot in the midbody (Fig. 1). Because there was no active bleeding, an intervention was deferred, and a biopsy sample was taken from lesion's edges. Initial histopathologic examination results were inconclusive, and deeper samples were required for analysis. Contrast-enhanced computed tomography of the abdomen showed an arterial-enhancing lesion (3.8 × 2.3 × 3 cm) seen on the anterior wall along the greater curvature. A feeding vessel originating from the short gastric artery, branch of splenic artery was identified (Fig. 2). Linear EUS showed a large, well-defined, hypoechoic tumor with increased vascularity and an irregular luminal surface. The lesion was seen within the submucosa with focal infiltration into the muscularis propria, where a large feeding vessel was observed entering into the lesion (Fig. 3).

Her case was discussed in a multidisciplinary meeting, and it was decided to pursue endoscopic submucosal dissection (ESD), with the patient’s approval secured. The procedure was performed with the patient under general anesthesia with carbon dioxide insufflation. Prophylactic antibiotic (cefoperazone plus sulbactam 1 gram) was administered before ESD.

Considering the high vascularity of the tumor and the presence of a large feeding vessel, severe bleeding during ESD was anticipated. Initially, glue embolization of the feeder vessel under EUS guidance was considered. However, it was avoided because of concerns that glue might hinder subsequent ESD. Instead, a pre-emptive epinephrine injection (1:10,000) was injected at the lesion's base near the feeder vessel under EUS guidance with a 19-gauge fine-needle aspiration needle. After we accessed the base, blood was aspirated to confirm proper needle positioning before injecting epinephrine. A total of 5 mL of diluted epinephrine was injected when aspiration did not yield blood. The first 2 mL was injected at the base near the feeder vessel, and 3 mL was injected while the needle was withdrawn. During and after the injection, heart rate and blood pressure were closely monitored. This approach allowed effective tamponade and vasoconstriction near the feeding vessel without the risks associated with direct intravascular injection. Direct intravascular injection of epinephrine into the feeder vessel was initially intended, but avoided for 2 reasons: (1) accurately targeting feeder vessel was technically challenging and (2) there were concerns about potential systemic effects, such as tachycardia and hypertension, associated with intravascular epinephrine injection.

**Figure 4:**
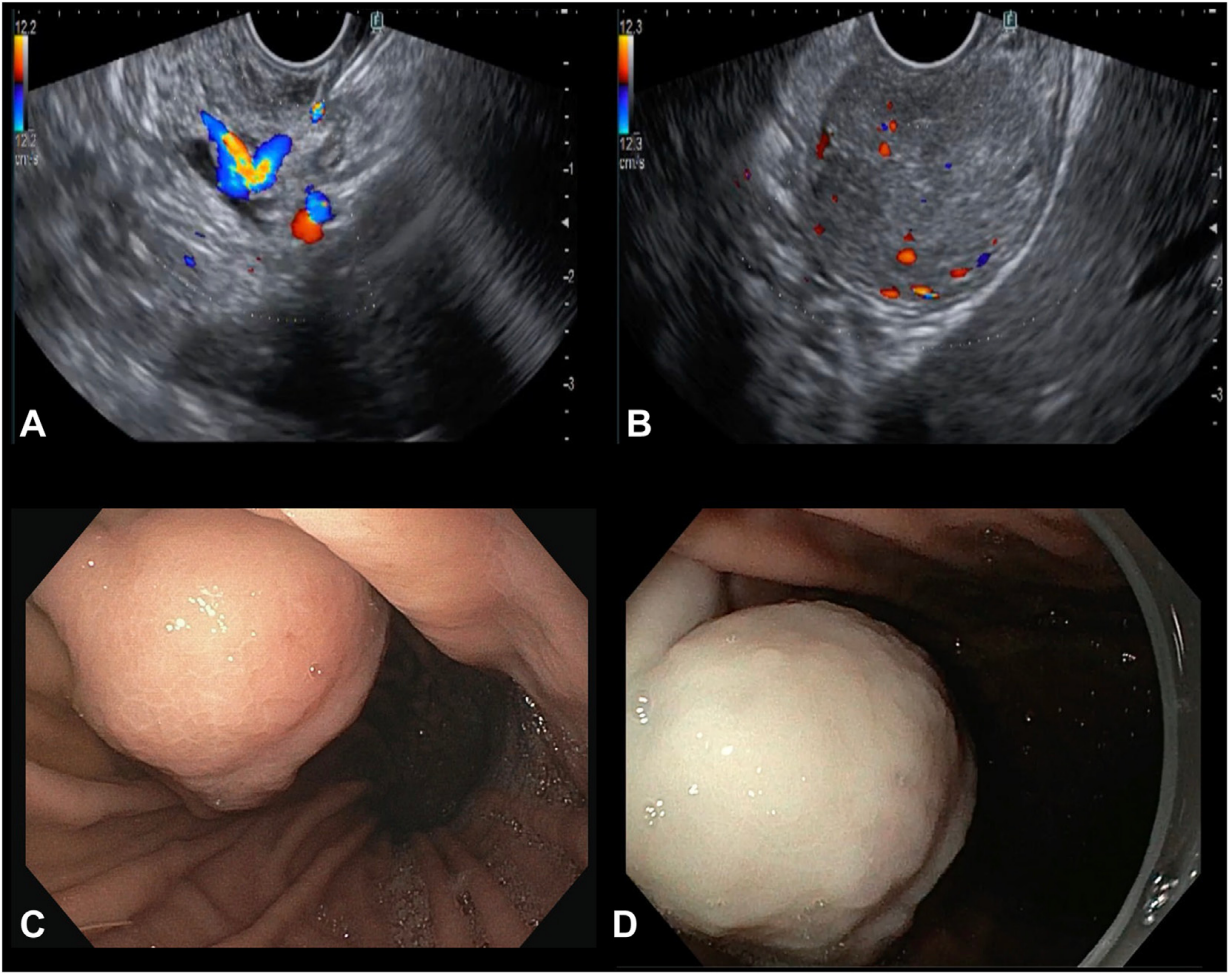
Pre-emptive EUS-guided epinephrine injection performed at the base into the feeder vessel. **A,** The feeder vessel was identified on EUS, and adjacent area was targeted with a 19-gauge needle, and diluted epinephrine injection (1:10,000) was administered. **B,** After epinephrine injection, a significant decrease in Doppler flow was noted. **C,** Gastroscopic view of the gastric lesion before epinephrine injection. **D,** Gastroscopic view of the gastric lesion postepinephrine injection, showing the lesion becoming pale.

**Figure 5:**
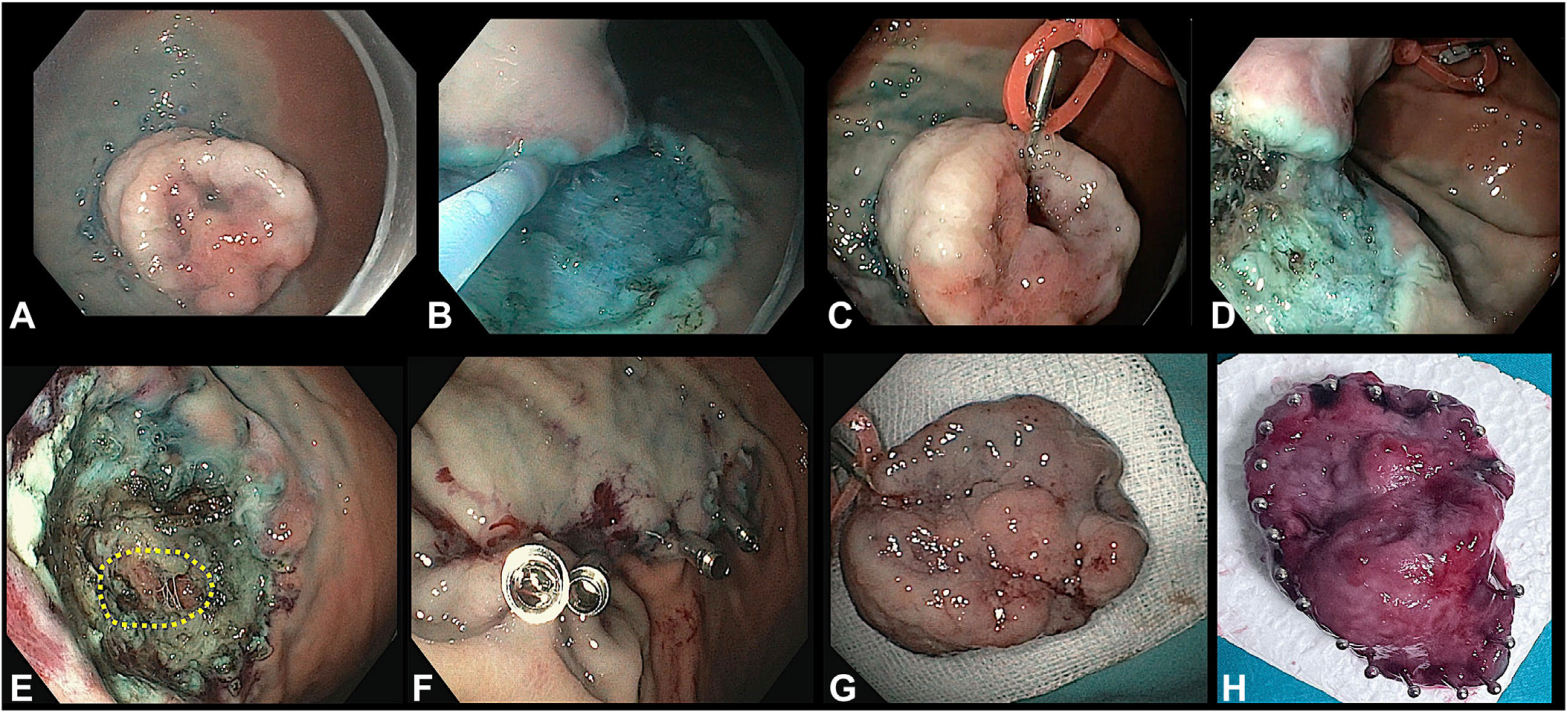
Endoscopic submucosal dissection performed for a large gastric subepithelial lesion. **A,** Markings were made around the lesion, and an incision was taken. **B,** The incision was deepened, and submucosal dissection was started by retroverting the gastroscope. **C,** A rubber band and clip were used for traction because of the difficulty in performing dissection underneath of the lesion. **D,** After rubber band and clip traction, submucosal fibers were well exposed. **E,** The entire lesion was removed in en bloc. The post-ESD base revealed defects in muscularis propria, with some serosal tissue also visible. **F,** The entire base was closed with 8 through-the-scope clips. **G,** Retrieved specimen with rubber band and clip attached. **H,** Mounted specimen measuring 40 mm in length and 28 mm in width.

The echoendoscope was then replaced with a gastroscope, and instantaneous pallor of the lesion was appreciated (Fig. 4). Circular markings were made around the lesion with a DualKnife J (Olympus, Tokyo, Japan). Submucosal elevation was performed at the marking site by injecting a diluted solution of methylene blue in saline. An incision was initially made at the proximal site, followed by a distal incision. After incision, submucosal dissection was started. Intermittently, an insulated-tip knife also was used during dissection. Because of the difficulty in performing dissection underneath the lesion, rubber band-clip traction was used to facilitate it. There were no major bleeding episodes observed during the procedure. However, few larger vessels that were visualized were prophylactically coagulated with hemostatic forceps. Notably, very few smaller vessels were encountered compared with typical gastric ESD cases. This reduction in visible vascularity is likely attributable to the vasoconstrictive effect of the pre-emptive EUS-guided epinephrine injection. The entire lesion was removed en bloc. After dissection, a full-thickness defect seen within the base. The whole base was closed with 8 through-the-scope clips (5 Klosure [Biorad Medisys, Pune, India] and 3 Ezclip [Olympus, Tokyo, Japan]) (Fig. 5, Video 1, available online at www.videogie.org). Total duration of procedure was 140 minutes, and there was no further need of epinephrine injection required during the procedure.

After the procedure, the patient was observed in the intensive care unit. A proton pump inhibitor infusion was initiated, and antibiotics were continued. Acetaminophen was administered for abdominal pain for 1 day. On the first procedure day, the findings of an oral contrast study ruled out leak, and patient was gradually started on diet. She was discharged in stable condition on day 3.

**Figure 6:**
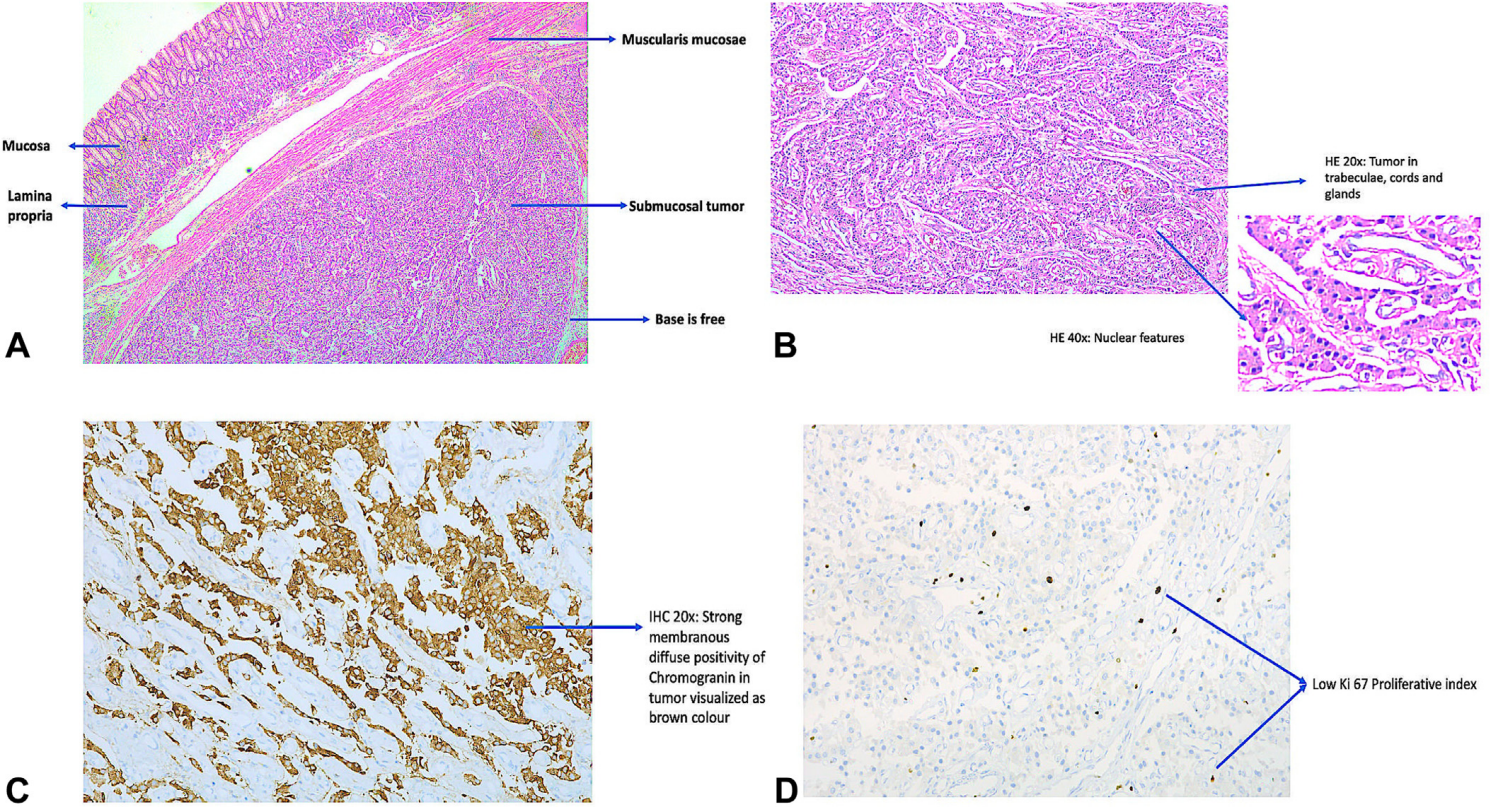
Histopathologic examination of resected gastric subepithelial lesion showed well-differentiated, grade I neuroendocrine tumor with Ki 67 index of <3%. **A,** Hematoxylin and eosin (H&E) stain, original magnification 10×, shows submucosal tumor with intact overlying mucosa normal and negative deep margin. **B,** H&E, original magnification 20×, shows a tumor composed of interlacing nests, cords, and trabeculae of uniform cells with round-to-oval nuclei and moderate eosinophilic cytoplasm. At original magnification 40×, the cells exhibit distinct nuclear features, including finely granular chromatin and inconspicuous nucleoli. **C,** Immunohistochemistry (IHC), original magnification 20×, shows strong diffuse positivity for chromogranin A in tumor cells. **D,** IHC, original magnification 20×, shows low Ki67 proliferative index (<3%).

**Figure 7:**
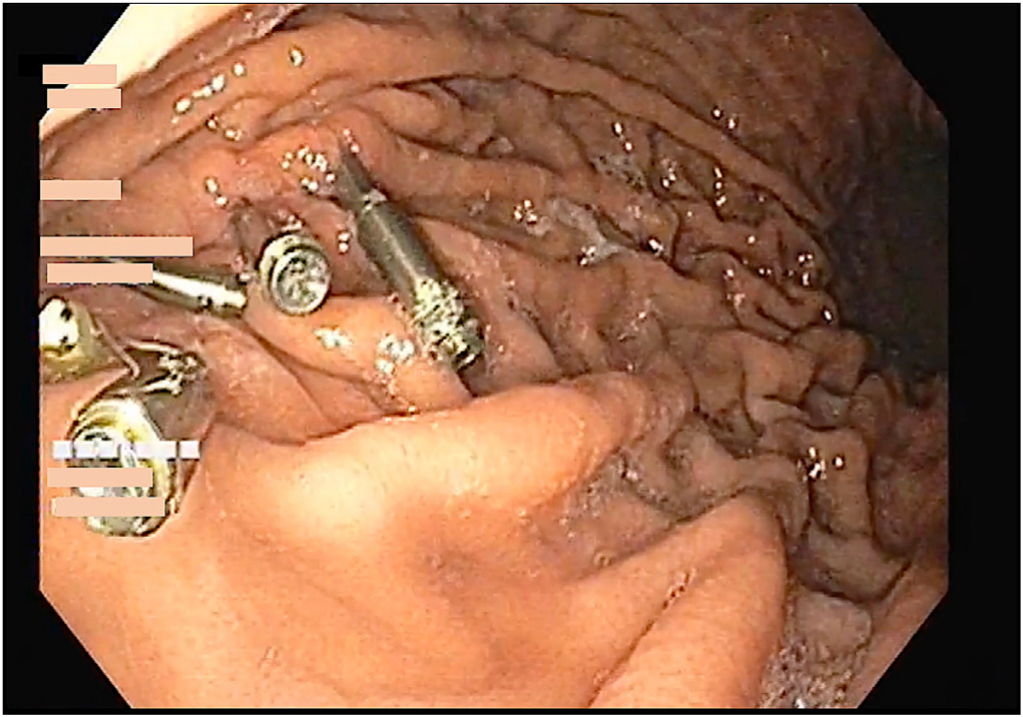
Repeat gastroscopy at 3-month follow-up showed scarring at previous endoscopic submucosal dissection site with 5 through-the-scope clips in situ.

Her histopathologic examination findings revealed a well-differentiated grade I neuroendocrine tumor (NET) with Ki 67 index <3%. There was no lymphovascular invasion, and the base and horizontal margins were free of tumor (Fig. 6). Gastrin levels were not assessed because of proton pump inhibitor use. In the absence of gastric atrophy with a single lesion, it was considered to be a type III low-grade gastric NET. Given the high metastatic risk, stringent follow-up was recommended. Repeat gastroscopy at 3-month follow-up showed scarring at the previous ESD site with 5 through-the-scope clips in situ (Fig. 7).

## Discussion

NETs are vascular tumors; therefore, bleeding during endotherapy is common.^1^^,^^2^ The majority of the bleeding episodes are mild-to-moderate and can be managed with conventional techniques. Torrential bleeding during ESD of gastric NET, although uncommon, can occur and may necessitate additional interventions.^3^ Our previous report demonstrated EUS-guided glue injection for managing torrential bleeding during gastric NET ESD.^3^ However, data on pre-emptive feeder vessel embolization before endotherapy remain limited. Few case reports demonstrated utility of interventional radiologist-guided embolization of the large feeder vessel prior to gastric polyp resection.^4^^,^^5^ To our knowledge, this is the first reported use of EUS-guided epinephrine injection to pre-emptively reduce lesion vascularity before gastric NET ESD (a representative image of the lesion is shown in Fig. 4). Further studies are needed to demonstrate safety and efficacy of this technique.

## Patient consent

The patient in this article has given written informed consent to publication of the case details.

## Disclosures

All authors disclosed no financial relationships.

## Supplementary data

Video 1 Shown is the use of preemptive EUS-guided epinephrine injection to minimize bleeding risk during endoscopic submucosal dissection.
